# Crystal structure and Hirshfeld surface analysis of dieth­yl (3a*S*,3*a*1*R*,4*S*,5*S*,6*R*,6a*S*,7*R*,9a*S*)-3a1,5,6,6a-tetra­hydro-1*H*,3*H*,4*H*,7*H*-3a,6:7,9a-di­epoxy­benzo[*de*]isochromene-4,5-di­carboxyl­ate

**DOI:** 10.1107/S2056989023010794

**Published:** 2024-01-01

**Authors:** Nurlana D. Sadikhova, Zeliha Atioğlu, Narmina A. Guliyeva, Alexandra G. Podrezova, Eugeniya V. Nikitina, Mehmet Akkurt, Ajaya Bhattarai

**Affiliations:** a Organic Chemistry Department, Baku State University, Az 1148 Baku, Azerbaijan; b Department of Aircraft Electrics and Electronics, School of Applied Sciences, Cappadocia University, Mustafapaşa, 50420 Ürgüp, Nevşehir, Türkiye; c Department of Organic Substances and Technology of High-Molecular Compounds, SRI "Geotechnological Problems of Oil, Gas and Chemistry", Azerbaijan State Oil and Industry University, Azadlig ave. 20, Az-1010 Baku, Azerbaijan; d Organic Chemistry Department, Faculty of Science, RUDN University, Miklukho-Maklaya St., 6, Moscow 117198, Russian Federation; e Department of Physics, Faculty of Sciences, Erciyes University, 38039 Kayseri, Türkiye; f Department of Chemistry, M.M.A.M.C (Tribhuvan University) Biratnagar, Nepal; Texas A & M University, USA

**Keywords:** crystal structure, (1*R*,4*S*)-7-oxabi­cyclo­[2.2.1]hept-2-ene, (1*S*,4*S*)-7-oxabi­cyclo­[2.2.1]hepta­ne, oxane, weak inter­actions, Hirshfeld surface analysis

## Abstract

In the title compound, C_18_H_22_O_7_, two hexane rings and an oxane ring are fused together. The two hexane rings tend toward a distorted boat conformation, while the tetra­hydro­furan and di­hydro­furan rings adopt envelope conformations. The oxane ring is puckered. The crystal structure features C—H⋯O hydrogen bonds, which link the mol­ecules into a three-dimensional network. According to a Hirshfeld surface study, H⋯H (60.3%) and O⋯H/H⋯O (35.3%) inter­actions are the most significant contributors to the crystal packing.

## Abstract 2

The crystal structure of the title compound features C—H⋯O hydrogen bonds, which link the mol­ecules into a three-dimensional network.

## 1. Chemical context

The inter­molecular Diels–Alder (DA) reaction of furans is a powerful tool in organic and medicinal chemistry, offering a versatile and efficient approach to the synthesis of complex mol­ecules with valuable applications (for reviews and books on the topic, see: Chen *et al.*, 2018 ▸; Winkler 1996 ▸; Parvatkar *et al.*, 2014 ▸; Shi & Wang, 2020 ▸; Hopf & Sherburn, 2012 ▸; Safavora *et al.*, 2019 ▸). The DA reaction of furans is typically carried out under thermal conditions, but the use of high pressure has emerged as a powerful tool for enhancing the reactivity and selectivity of this reaction. High pressure can significantly lower the activation energy of the DA reaction, leading to faster reaction rates and improved yields (see reviews by Rulev & Zubkov, 2022 ▸; Schettino & Bini, 2007 ▸). On the other hand, by the attachment of functional groups, the DA reaction products can participate in various sorts of inter­molecular inter­actions with inter­esting coordination, supra­molecular, catalytic and solvatochromic properties (Gurbanov *et al.*, 2020*a* ▸,*b* ▸; Khalilov *et al.*, 2021 ▸; Mahmoudi *et al.*, 2017*a* ▸,*b* ▸; Mahmudov *et al.*, 2015 ▸). For example, attachment of carb­oxyl­ate groups to organic mol­ecules can create coordination sites and inter­esting supra­molecular architectures involving monomeric, oligomeric or polymeric subunits in metal complexes, which affects their catalytic activity (Gurbanov *et al.*, 2022*a* ▸,*b* ▸; Ma *et al.*, 2017 ▸, 2021 ▸; Shikhaliyev *et al.*, 2019 ▸). The present work showcases a facile methodology for the synthesis of compound **1a** from a simple furan derivative and diethyl fumarate under high-pressure conditions. It is noteworthy that while several methods for the preparation of similar structures using more reactive dienophiles have been documented in the literature (Borisova *et al.*, 2018*a* ▸,*b* ▸; Kvyatkovskaya *et al.*, 2021*a* ▸,*b* ▸), this represents the first instance of such a reaction where thermal activation alone is insufficient to drive the transformation. 
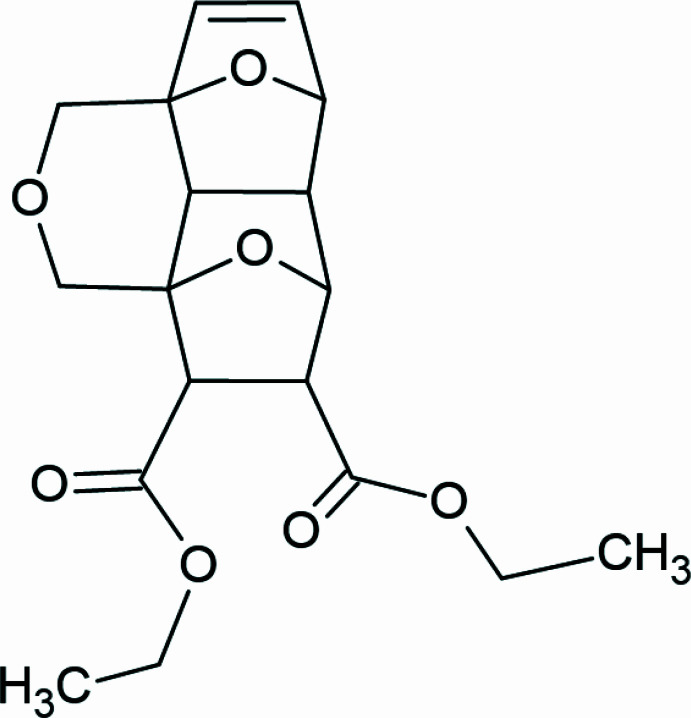


## 2. Structural commentary

**Figure 1:**
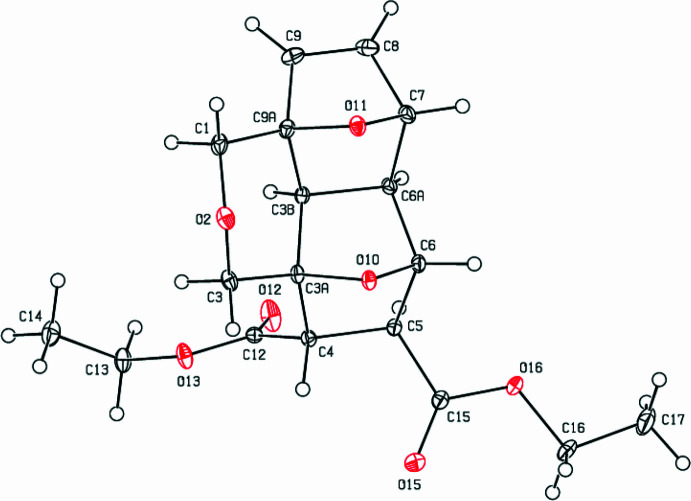
The mol­ecular structure of the title complex with displacement ellipsoids for the non-hydrogen atoms drawn at the 50% probability level.

In the title compound, (Fig. 1 ▸), the (1*R*,4*S*)-7-oxabi­cyclo­[2.2.1]hept-2-ene (O11/C3*B*/C6*A*/C7–C9/C9*A*), (1*S*,4*S*)-7-oxabi­cyclo­[2.2.1]heptane (O10/C3*A*/C3*B*/C4–C6/C6*A*) and and oxane (C1/O2/C3/C3*A*/C3*B*/C9*A*) rings are fused together. The hexane ring (C3*B*/C6*A*/C7–C9/C9*A*) tends towards a distorted boat conformation [the puckering parameters (Cremer & Pople, 1975 ▸) are *Q*_T_ = 1.0005 (15) Å, θ = 89.65 (9)° and φ = 300.58 (8)°], while the tetra­hydro­furan (C3*B*/C6*A*/C7/O11/C9*A*) and di­hydro­furan (C7–C9/C9*A*/O11) rings adopt envelope conformations, with puckering parameters *Q*(2) = 0.5625 (13) Å, φ(2) = 1.70 (14)° and *Q*(2) = 0.5143 (13) Å, φ(2) = 179.57 (17)°, respectively. The hexane ring (C3*A*/C3*B*/C4–C6/C6*A*) tends towards a distorted boat conformation [puckering parameters *Q*_T_ = 0.9758 (14) Å, θ = 91.04 (8)° and φ = 2.99 (8)°], while the tetra­hydro­furan rings (C3*A*/C4–C6/O10 and C6/C6*A*/C3*B*/C3*A*/O10) adopt envelope conformations, with puckering parameters *Q*(2) = 0.5784 (13) Å, φ(2) = 185.10 (14)° and Q(2) = 0.5235 (13) Å, φ(2) = 357.36 (15)°, respectively. The oxane ring (C3*A*/C3*B*/C9*A*/C1/O2/C3) is puckered with puckering parameters *Q*_T_ = 0.5125 (14) Å, θ = 7.89 (15)° and φ = 3.1 (12)°. The C*3A*—C4—C12—O12, C3*A*—C4—C12—O13, C4—C12—O13—C13, C6—C5—C15—O15, C6—C5—C15—O16 and C5—C15—O16—C16 torsion angles are 107.12 (15), −72.14 (13), −177.31 (11), 120.28 (15), −60.17 (14) and 178.70 (11)°, respectively. The geometric parameters of the title compound are normal and comparable to those of related compounds listed in the *Database survey* section.

## 3. Supra­molecular features and Hirshfeld surface analysis

**Figure 2:**
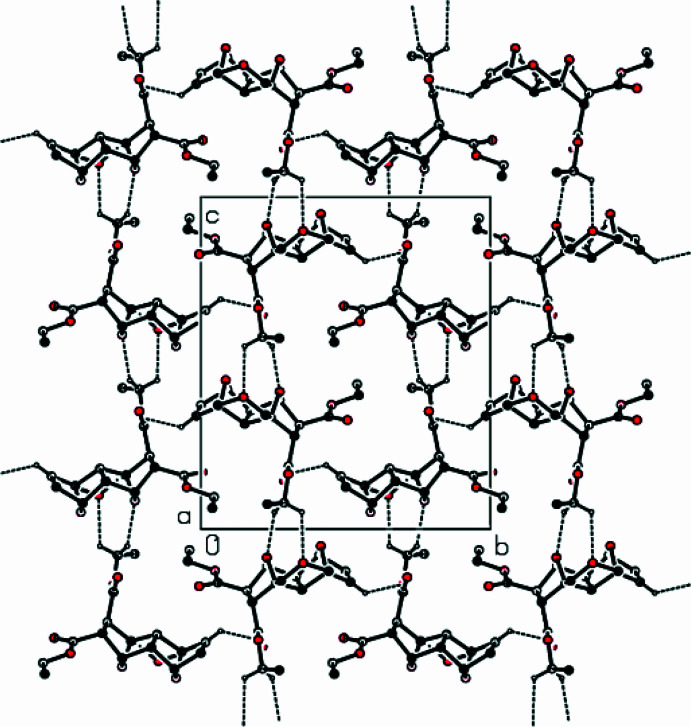
A packing diagram of the title complex, showing the C—H⋯O inter­actions along the *a* axis as dashed lines.

The crystal structure of the title compound is stabilized by C—H⋯O hydrogen bonds, forming a three-dimensional network (Table 1 ▸; Figs. 2 ▸, 3 ▸ and 4 ▸). C—H⋯π and π–π inter­actions are not observed in the structure.

**Figure 5:**
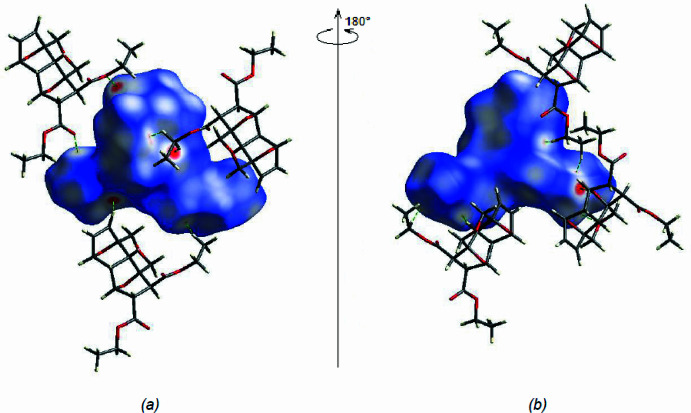
(*a*) Front and (*b*) back sides of the three-dimensional Hirshfeld surface of the title compound mapped over *d*_norm_, with a fixed colour scale of −0.1982 to +1.2419 a.u.

**Figure 6:**
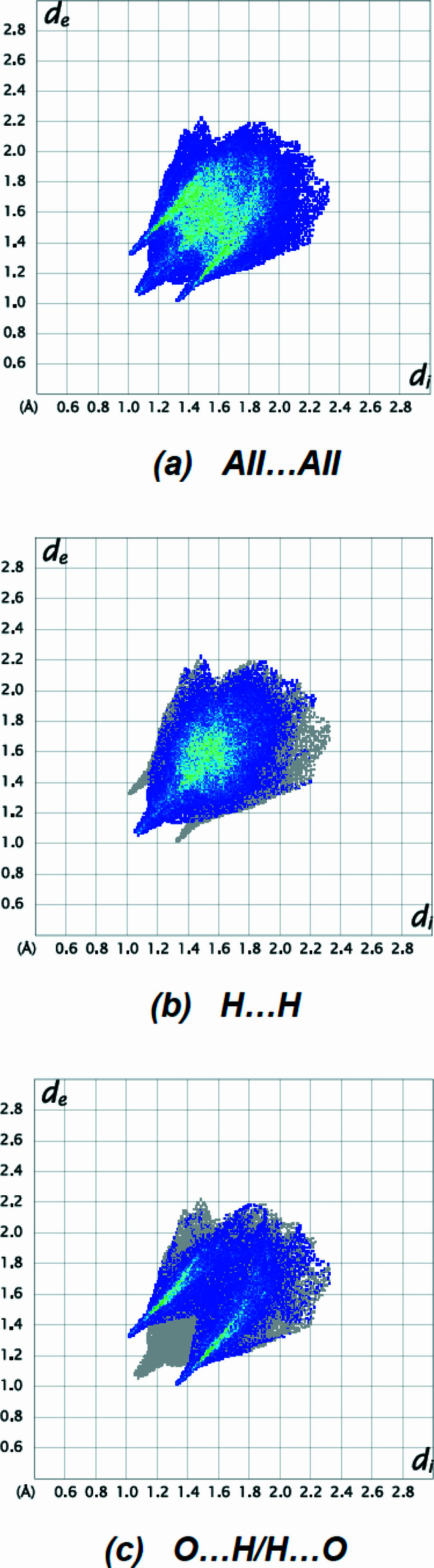
The two-dimensional fingerprint plots of the title compound, showing (*a*) all inter­actions, and delineated into (*b*) H⋯H and (*c*) O⋯H/H⋯O inter­actions. [*d*_e_ and *d*_i_ represent the distances from a point on the Hirshfeld surface to the nearest atoms outside (external) and inside (inter­nal) the surface, respectively].

*Crystal Explorer 17.5* (Spackman *et al.*, 2021 ▸) was used to generate Hirshfeld surfaces and two-dimensional fingerprint plots in order to qu­antify the inter­molecular inter­actions in the crystal. The Hirshfeld surfaces were mapped over *d*_norm_ (Fig. 5 ▸). The inter­actions listed in Table 2 ▸ play a key role in the mol­ecular packing of the title compound. The most important inter­atomic contact is H⋯H as it makes the highest contribution to the crystal packing (60.2%, Fig. 6 ▸ *b*). The other major contributor is the O⋯H/H⋯O (35.4%, Fig. 6 ▸ *c*) inter­action. Other, smaller contributions are made by C⋯H/H⋯C (3.9%), O⋯O (0.3%), C⋯C (0.2%) and O⋯C/C⋯O (0.1%) inter­actions.

## 4. Database survey

Four related compounds were found in a search of the Cambridge Structural Database (CSD, version 5.42, update of September 2021; Groom *et al.*, 2016 ▸), *viz. N*-carbamo­thioyl­amino-7-oxabi­cyclo­[2.2.1]hept-5-ene-2,3-dicarboximide (CSD refcode WAFPOK; Li, 2010 ▸), {3-hy­droxy­methyl-1-[2-(3-methoxy­phen­yl)eth­yl]-7-oxabi­cyclo­(2.2.1)hept-5-en-2-yl}methanol (SIMPUA; Wang & Peng, 2007 ▸), (1*SR*,2*SR*,4*SR*)-7-oxabi­cyclo­(2.2.1)hept-5-ene-2-carb­oxy­lic acid (ETEYEH; Gartenmann Dickson *et al.*, 2004 ▸) and (1S*,2R*,5S*,6S*,7R*)-5-hy­droxy-4-(4-meth­oxy­phen­yl)-10-oxa-4-aza­tri­cyclo­(5.2.1.02,6)dec-8-en-one (DIWLEB; Gökçe *et al.*, 2008 ▸).

The compound WAFPOK comprises a racemic mixture of chiral mol­ecules containing four stereogenic centres. The cyclo­hexane ring tends towards a boat conformation, while the tetra­hydro­furan and di­hydro­furan rings adopt envelope conformations. The dihedral angle between the thio­semicarbazide fragment and the fused-ring system is 77.20 (10)°. The crystal structure is stabilized by two inter­molecular N—H⋯O hydrogen bonds. SIMPUA is an oxabi­cyclo­[2.2.1]hept-5- ene with two exo-oriented hy­droxy­methyl groups, which are not parallel to each other. The mol­ecules are linked to each other by hydrogen bonds, resulting in a supra­molecular network. Inter­molecular O—H⋯O hydrogen bonding is observed between the hydroxyl groups. In ETEYEH, the mol­ecules are connected by O—H⋯O hydrogen bonds, forming centrosymmetric dimers. The structure of DIWLEB comprises a racemic mixture of chiral mol­ecules containing five stereogenic centres. The cyclo­hexane ring tends towards a boat conformation and the two tetra­hydro­furan rings adopt envelope conformations. Mol­ecules are linked into sheets parallel to (100) by a combination of O—H⋯O, C—H⋯O and C—H⋯π inter­actions, leading to a di-periodic supra­molecular structure.

## 5. Synthesis and crystallization

**Figure 7:**
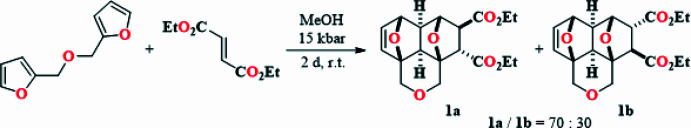
Reaction mechanism.

A solution of diethyl fumarate (850 mg, 4.95 mmol, 1.1 equiv) and difurfuryl ether (800 mg, 4.5 mmol) in methanol (21 mL) was placed in a Teflon ampoule. The reaction mixture was then held at 15 kbar and r.t. for two days in a piston-cylinder type steel pressure chamber. The obtained methanol solution was concentrated *in vacuo*. The resulting light-yellow oil was solidified in hexane and then recrystallized from ethyl acetate to isolate diastereomer **1a** exclusively (Fig. 7 ▸). The residue was filtered off and dried under reduced pressure in a vacuum desiccator to constant weight, yielding the target product as white crystals. White crystals, 0.32 g, 0.94 mmol, yield is 21%, *R*_f_ = 0.7 (‘Sorbfil’ plates for thin-layer chromatography, CHCl_3_); mp: 418.1–419.1 K. A single-crystal of compound **1a** was obtained by slow evaporation from ethyl acetate at 298 K.

^1^H NMR (700 MHz, CDCl_3_, 298 K) *δ* 6.40 (*d*, *J* = 5.7 Hz, 1H, CH=CH), 6.20 (*d*, *J* = 5.7 Hz, 1H, CH=CH), 5.02 (*s*, 1H, CH), 4.83 (*s*, 1H, CH), 4.38 (*d*, *J* = 12.8 Hz, 1H, from CH_2_), 4.27 (*d*, *J* = 12.9 Hz, 1H, from CH_2_), 4.23–4.13 (*m*, 4H, 2OCH_2_CH_3_), 4.02 (*d*, *J* = 12.9 Hz, 1H, from CH_2_), 3.89 (*d*, *J* = 12.8 Hz, 1H, from CH_2_), 3.19 (*d*, *J* = 5.2 Hz, 1H, CH), 3.12 (*d*, *J* = 5.2 Hz, 1H, CH), 2.13 (*d*, *J* = 6.4 Hz, 1H, CH), 1.75 (*d*, *J* = 6.3 Hz, 1H, CH), 1.36–1.14 (*m*, 6H, 2OCH_2_CH_3_) . ^13^C NMR (175 MHz, CDCl_3_, 298 K) *δ* 171.69, 170.50, 138.05, 136.58, 84.04, 82.64, 82.26, 81.65, 66.67, 66.29, 61.52 (2C), 53.12, 52.70, 49.90, 43.15, 14.34, 14.25. IR *ν*_max_/cm^−1^ (tablet KBr): 3440, 2982, 2950, 2910, 2859, 1728, 1473, 1311, 1211, 1174, 1093, 1029, 973, 912, 856, 697. HRMS (ESI-TOF): calculated for C_18_H_23_O_7_ [*M* + H]^+^ 351.1443; found 351.1440.

## 6. Refinement

Crystal data, data collection and structure refinement details are summarized in Table 3 ▸. C-bound H atoms were included in the refinement using the riding-model approximation with C—H distances of 0.95–1.00 Å, and with *U*_iso_(H) = 1.2 or 1.5*U*_eq_(C). Two reflections (0 1 1 and 1 1 0), affected by the incident beam-stop, were omitted in the final cycles of refinement.

## Supplementary Material

Crystal structure: contains datablock(s) I. DOI: 10.1107/S2056989023010794/jy2042sup1.cif

Structure factors: contains datablock(s) I. DOI: 10.1107/S2056989023010794/jy2042Isup2.hkl

CCDC reference: 2319519

Additional supporting information:  crystallographic information; 3D view; checkCIF report

## Figures and Tables

**Figure 3:**
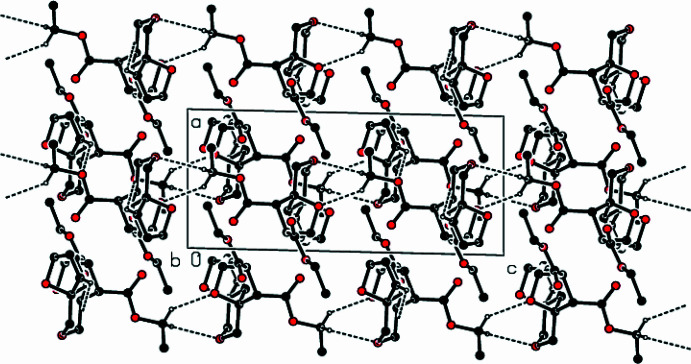
View of the crystal structure of the title complex, along the *b* axis; the same inter­actions are as in Fig. 2 ▸.

**Figure 4:**
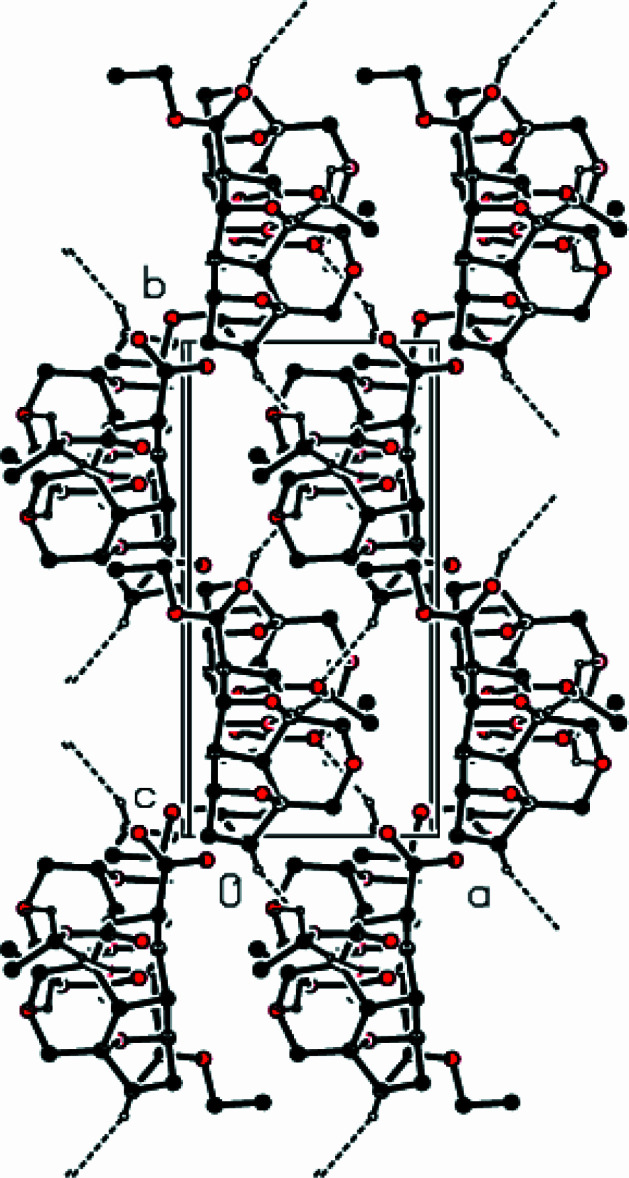
View of the crystal structure of the title complex, along the *c* axis; the same inter­actions are as in Fig. 2 ▸.

**Table 1:** Hydrogen-bond geometry (Å, °)

*D*—H⋯*A*	*D*—H	H⋯*A*	*D*⋯*A*	*D*—H⋯*A*
C3—H3*A*⋯O13	0.99	2.58	3.1604 (17)	118
C9—H9*A*⋯O13^i^	0.95	2.47	3.1692 (17)	131
C13—H13*A*⋯O2^ii^	0.99	2.58	3.1905 (17)	120
C13—H13*B*⋯O10^ii^	0.99	2.41	3.2193 (17)	139
C14—H14*C*⋯O15^i^	0.98	2.64	3.5669 (19)	158

Symmetry codes: (i) 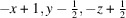 ; (ii) 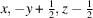 .

**Table 2:** Summary of short inter­atomic contacts (Å) in the title compound

Contact	Distance	Symmetry operation
H3*A*⋯H6*AA*	2.49	1 + *x*, *y*, *z*
O10⋯H13*B*	2.41	*x*, 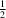 − *y*, 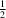 + *z*
H16*B*⋯H16*B*	2.57	−*x*, 1 − *y*, 1 − *z*
H1*A*⋯O11	2.73	1 − *x*, −*y*, 1 − *z*
H17*A*⋯H13*B*	2.29	−*x*, 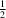 + *y*, 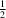 − *z*
O13⋯H9*A*	2.47	1 − *x*, 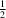 + *y*, 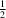 − *z*
H7*A*⋯C8	3.02	−*x*, −*y*, 1 − *z*
H6*A*⋯H14*A*	2.50	−1 + *x*, 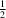 − *y*, 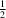 + *z*

**Table 3:** Experimental details

Crystal data	
Chemical formula	C_18_H_22_O_7_
*M*_r_	350.35
Crystal system, space group	Monoclinic, *P*2_1_/*c*
Temperature (K)	100
*a*, *b*, *c* (Å)	7.1566 (4), 14.1907 (9), 16.2737 (10)
β (°)	91.329 (2)
*V* (Å^3^)	1652.27 (17)
*Z*	4
Radiation type	Mo *K*α
μ (mm^−1^)	0.11
Crystal size (mm)	0.40 × 0.36 × 0.34

Data collection	
Diffractometer	Bruker Kappa APEXII area-detector diffractometer
Absorption correction	Multi-scan (*SADABS*; Krause *et al.*, 2015 ▸).
*T*_min_, *T*_max_	0.941, 1.000
No. of measured, independent and observed [*I* > 2σ(*I*)] reflections	28552, 4921, 3615
*R*_int_	0.051
(sin θ/λ)_max_ (Å^−1^)	0.709

Refinement	
*R*[*F* ^2^ > 2σ(*F* ^2^)], *wR*(*F* ^2^), *S*	0.046, 0.119, 1.03
No. of reflections	4921
No. of parameters	226
H-atom treatment	H-atom parameters constrained
Δρ_max_, Δρ_min_ (e Å^−3^)	0.35, −0.30

Computer programs: *APEX3* and *SAINT* (Bruker, 2018 ▸), *SHELXT2014/5* (Sheldrick, 2015*a* ▸), *SHELXL2018/3* (Sheldrick, 2015*b* ▸), *ORTEP-3 for Windows* (Farrugia, 2012 ▸) and *PLATON* (Spek, 2020 ▸).

## supplementary crystallographic information

### Crystal data

**Table:**

C_18_H_22_O_7_	*F*(000) = 744
*M**_r_* = 350.35	*D*_x_ = 1.408 Mg m^−^^3^
Monoclinic, *P*2_1_/*c*	Mo *K*α radiation, λ = 0.71073 Å
*a* = 7.1566 (4) Å	Cell parameters from 5852 reflections
*b* = 14.1907 (9) Å	θ = 2.5–29.9°
*c* = 16.2737 (10) Å	µ = 0.11 mm^−^^1^
β = 91.329 (2)°	*T* = 100 K
*V* = 1652.27 (17) Å^3^	Bulk, colourless
*Z* = 4	0.40 × 0.36 × 0.34 mm

### Data collection

**Table:**

Bruker KAPPA APEXII area-detector diffractometer	3615 reflections with *I* > 2σ(*I*)
φ and ω scans	*R*_int_ = 0.051
Absorption correction: multi-scan (SADABS; Krause *et al.*, 2015).	θ_max_ = 30.3°, θ_min_ = 2.9°
*T*_min_ = 0.941, *T*_max_ = 1.000	*h* = −10→10
28552 measured reflections	*k* = −20→18
4921 independent reflections	*l* = −23→22

### Refinement

**Table:**

Refinement on *F*^2^	0 restraints
Least-squares matrix: full	Hydrogen site location: inferred from neighbouring sites
*R*[*F*^2^ > 2σ(*F*^2^)] = 0.046	H-atom parameters constrained
*wR*(*F*^2^) = 0.119	*w* = 1/[σ^2^(*F*_o_^2^) + (0.0541*P*)^2^ + 0.5731*P*] where *P* = (*F*_o_^2^ + 2*F*_c_^2^)/3
*S* = 1.03	(Δ/σ)_max_ < 0.001
4921 reflections	Δρ_max_ = 0.35 e Å^−^^3^
226 parameters	Δρ_min_ = −0.30 e Å^−^^3^

### Special details

**Table:**

Geometry. All esds (except the esd in the dihedral angle between two l.s. planes) are estimated using the full covariance matrix. The cell esds are taken into account individually in the estimation of esds in distances, angles and torsion angles; correlations between esds in cell parameters are only used when they are defined by crystal symmetry. An approximate (isotropic) treatment of cell esds is used for estimating esds involving l.s. planes.

### Fractional atomic coordinates and isotropic or equivalent isotropic displacement parameters (Å^2^)

**Table:**

	*x*	*y*	*z*	*U*_iso_*/*U*_eq_	
O2	0.65003 (13)	0.14690 (7)	0.39784 (6)	0.0185 (2)	
O10	0.32231 (13)	0.27216 (6)	0.41424 (6)	0.01429 (19)	
O11	0.28123 (13)	0.08569 (7)	0.44881 (6)	0.0166 (2)	
O12	0.20009 (15)	0.28745 (9)	0.14509 (7)	0.0289 (3)	
O13	0.51089 (13)	0.30191 (7)	0.16601 (6)	0.0202 (2)	
O15	0.20749 (15)	0.50604 (7)	0.32809 (7)	0.0275 (3)	
O16	−0.06545 (14)	0.45134 (7)	0.37426 (6)	0.0203 (2)	
C1	0.56703 (19)	0.05898 (10)	0.37326 (9)	0.0190 (3)	
H1A	0.604785	0.009304	0.412996	0.023*	
H1B	0.613288	0.040808	0.318629	0.023*	
C3B	0.28585 (17)	0.15148 (9)	0.31737 (8)	0.0127 (2)	
H3BA	0.276226	0.137729	0.257158	0.015*	
C3A	0.39716 (17)	0.24133 (9)	0.33732 (8)	0.0129 (2)	
C3	0.60532 (18)	0.22335 (10)	0.34389 (8)	0.0167 (3)	
H3A	0.652818	0.209205	0.288583	0.020*	
H3B	0.668881	0.281058	0.364234	0.020*	
C4	0.32813 (18)	0.32304 (9)	0.28048 (8)	0.0132 (2)	
H4A	0.406592	0.379971	0.292317	0.016*	
C5	0.13032 (18)	0.34034 (9)	0.31390 (8)	0.0139 (2)	
H5A	0.033387	0.321017	0.271973	0.017*	
C6	0.12801 (18)	0.27188 (9)	0.38872 (8)	0.0144 (3)	
H6A	0.040849	0.291342	0.432769	0.017*	
C6A	0.09390 (18)	0.17152 (9)	0.35618 (8)	0.0141 (2)	
H6AA	−0.012435	0.168001	0.315333	0.017*	
C7	0.08827 (19)	0.09147 (9)	0.42186 (9)	0.0173 (3)	
H7A	−0.003793	0.100706	0.466452	0.021*	
C8	0.0674 (2)	0.00021 (10)	0.37333 (10)	0.0224 (3)	
H8A	−0.041834	−0.037460	0.367396	0.027*	
C9A	0.35656 (18)	0.06594 (9)	0.36921 (8)	0.0155 (3)	
C9	0.2335 (2)	−0.01604 (9)	0.34091 (9)	0.0210 (3)	
H9A	0.267854	−0.067681	0.307329	0.025*	
C12	0.33375 (18)	0.30231 (9)	0.18944 (8)	0.0136 (2)	
C13	0.5500 (2)	0.28738 (11)	0.07909 (8)	0.0200 (3)	
H13A	0.568211	0.348732	0.051437	0.024*	
H13B	0.444317	0.254289	0.051389	0.024*	
C14	0.7242 (2)	0.22918 (11)	0.07489 (10)	0.0259 (3)	
H14A	0.754364	0.218155	0.017244	0.039*	
H14B	0.827848	0.262688	0.102366	0.039*	
H14C	0.704464	0.168635	0.102318	0.039*	
C15	0.10027 (19)	0.44187 (9)	0.33870 (8)	0.0159 (3)	
C16	−0.1128 (2)	0.54538 (10)	0.40327 (9)	0.0216 (3)	
H16A	−0.109745	0.591211	0.357452	0.026*	
H16B	−0.022582	0.565926	0.446699	0.026*	
C17	−0.3058 (2)	0.53933 (13)	0.43679 (13)	0.0369 (4)	
H17A	−0.343372	0.601375	0.457067	0.055*	
H17B	−0.393486	0.518921	0.393188	0.055*	
H17C	−0.306732	0.493754	0.482017	0.055*	

### Atomic displacement parameters (Å^2^)

**Table:**

	*U*^11^	*U*^22^	*U*^33^	*U*^12^	*U*^13^	*U*^23^
O2	0.0118 (4)	0.0270 (5)	0.0167 (5)	0.0022 (4)	−0.0019 (4)	0.0041 (4)
O10	0.0130 (4)	0.0189 (4)	0.0110 (4)	0.0006 (3)	0.0019 (3)	−0.0018 (3)
O11	0.0142 (4)	0.0204 (5)	0.0150 (5)	0.0032 (4)	0.0017 (4)	0.0033 (4)
O12	0.0162 (5)	0.0536 (7)	0.0168 (5)	0.0001 (5)	−0.0023 (4)	−0.0039 (5)
O13	0.0136 (5)	0.0351 (6)	0.0121 (5)	−0.0026 (4)	0.0021 (4)	−0.0042 (4)
O15	0.0267 (6)	0.0169 (5)	0.0394 (7)	−0.0021 (4)	0.0097 (5)	−0.0038 (4)
O16	0.0207 (5)	0.0145 (4)	0.0260 (6)	0.0047 (4)	0.0069 (4)	−0.0019 (4)
C1	0.0142 (6)	0.0225 (7)	0.0204 (7)	0.0070 (5)	0.0014 (5)	0.0027 (5)
C3B	0.0105 (5)	0.0150 (6)	0.0126 (6)	0.0016 (4)	0.0000 (5)	−0.0006 (4)
C3A	0.0113 (6)	0.0171 (6)	0.0102 (6)	0.0008 (4)	0.0015 (5)	−0.0012 (4)
C3	0.0108 (6)	0.0244 (7)	0.0148 (6)	0.0006 (5)	0.0006 (5)	0.0034 (5)
C4	0.0129 (6)	0.0148 (6)	0.0120 (6)	−0.0005 (4)	0.0017 (5)	−0.0005 (4)
C5	0.0125 (6)	0.0143 (6)	0.0149 (6)	0.0014 (5)	0.0019 (5)	−0.0010 (5)
C6	0.0115 (6)	0.0164 (6)	0.0153 (6)	0.0024 (5)	0.0028 (5)	0.0006 (5)
C6A	0.0103 (6)	0.0147 (6)	0.0175 (6)	0.0011 (4)	0.0016 (5)	0.0027 (5)
C7	0.0117 (6)	0.0178 (6)	0.0224 (7)	0.0011 (5)	0.0023 (5)	0.0043 (5)
C8	0.0204 (7)	0.0155 (6)	0.0310 (8)	−0.0027 (5)	−0.0031 (6)	0.0049 (6)
C9A	0.0143 (6)	0.0164 (6)	0.0158 (6)	0.0037 (5)	0.0005 (5)	0.0003 (5)
C9	0.0245 (7)	0.0132 (6)	0.0251 (7)	0.0030 (5)	−0.0033 (6)	0.0009 (5)
C12	0.0146 (6)	0.0126 (6)	0.0134 (6)	0.0008 (4)	0.0007 (5)	0.0022 (4)
C13	0.0203 (7)	0.0295 (7)	0.0103 (6)	0.0035 (6)	0.0025 (5)	−0.0019 (5)
C14	0.0276 (8)	0.0308 (8)	0.0194 (7)	0.0090 (6)	0.0048 (6)	−0.0007 (6)
C15	0.0180 (6)	0.0166 (6)	0.0132 (6)	0.0035 (5)	0.0008 (5)	0.0006 (5)
C16	0.0267 (7)	0.0154 (6)	0.0226 (7)	0.0093 (5)	0.0013 (6)	−0.0026 (5)
C17	0.0280 (9)	0.0320 (9)	0.0512 (12)	0.0115 (7)	0.0102 (8)	−0.0112 (8)

### Geometric parameters (Å, º)

**Table:**

O2—C3	1.4274 (16)	C5—C15	1.5130 (18)
O2—C1	1.4347 (17)	C5—C6	1.5580 (18)
O10—C3A	1.4410 (15)	C5—H5A	1.0000
O10—C6	1.4420 (15)	C6—C6A	1.5370 (18)
O11—C7	1.4414 (16)	C6—H6A	1.0000
O11—C9A	1.4420 (16)	C6A—C7	1.5609 (18)
O12—C12	1.2035 (16)	C6A—H6AA	1.0000
O13—C12	1.3323 (16)	C7—C8	1.522 (2)
O13—C13	1.4630 (16)	C7—H7A	1.0000
O15—C15	1.2058 (17)	C8—C9	1.332 (2)
O16—C15	1.3384 (16)	C8—H8A	0.9500
O16—C16	1.4581 (16)	C9A—C9	1.5241 (19)
C1—C9A	1.5094 (18)	C9—H9A	0.9500
C1—H1A	0.9900	C13—C14	1.498 (2)
C1—H1B	0.9900	C13—H13A	0.9900
C3B—C3A	1.5341 (18)	C13—H13B	0.9900
C3B—C6A	1.5515 (17)	C14—H14A	0.9800
C3B—C9A	1.5561 (18)	C14—H14B	0.9800
C3B—H3BA	1.0000	C14—H14C	0.9800
C3A—C3	1.5127 (17)	C16—C17	1.499 (2)
C3A—C4	1.5565 (18)	C16—H16A	0.9900
C3—H3A	0.9900	C16—H16B	0.9900
C3—H3B	0.9900	C17—H17A	0.9800
C4—C12	1.5120 (18)	C17—H17B	0.9800
C4—C5	1.5478 (18)	C17—H17C	0.9800
C4—H4A	1.0000		
			
C3—O2—C1	113.82 (10)	C3B—C6A—H6AA	112.8
C3A—O10—C6	97.15 (9)	C7—C6A—H6AA	112.8
C7—O11—C9A	96.51 (10)	O11—C7—C8	101.02 (10)
C12—O13—C13	118.82 (11)	O11—C7—C6A	102.12 (10)
C15—O16—C16	116.54 (11)	C8—C7—C6A	105.52 (11)
O2—C1—C9A	111.20 (10)	O11—C7—H7A	115.4
O2—C1—H1A	109.4	C8—C7—H7A	115.4
C9A—C1—H1A	109.4	C6A—C7—H7A	115.4
O2—C1—H1B	109.4	C9—C8—C7	105.96 (12)
C9A—C1—H1B	109.4	C9—C8—H8A	127.0
H1A—C1—H1B	108.0	C7—C8—H8A	127.0
C3A—C3B—C6A	102.82 (10)	O11—C9A—C1	111.53 (11)
C3A—C3B—C9A	111.90 (10)	O11—C9A—C9	101.20 (11)
C6A—C3B—C9A	101.66 (10)	C1—C9A—C9	122.17 (11)
C3A—C3B—H3BA	113.2	O11—C9A—C3B	102.22 (10)
C6A—C3B—H3BA	113.1	C1—C9A—C3B	112.73 (11)
C9A—C3B—H3BA	113.1	C9—C9A—C3B	104.67 (11)
O10—C3A—C3	112.03 (10)	C8—C9—C9A	105.25 (12)
O10—C3A—C3B	103.56 (10)	C8—C9—H9A	127.4
C3—C3A—C3B	112.36 (10)	C9A—C9—H9A	127.4
O10—C3A—C4	99.83 (9)	O12—C12—O13	125.17 (12)
C3—C3A—C4	117.78 (11)	O12—C12—C4	125.66 (12)
C3B—C3A—C4	109.72 (10)	O13—C12—C4	109.17 (11)
O2—C3—C3A	112.21 (11)	O13—C13—C14	107.45 (12)
O2—C3—H3A	109.2	O13—C13—H13A	110.2
C3A—C3—H3A	109.2	C14—C13—H13A	110.2
O2—C3—H3B	109.2	O13—C13—H13B	110.2
C3A—C3—H3B	109.2	C14—C13—H13B	110.2
H3A—C3—H3B	107.9	H13A—C13—H13B	108.5
C12—C4—C5	114.86 (11)	C13—C14—H14A	109.5
C12—C4—C3A	114.96 (10)	C13—C14—H14B	109.5
C5—C4—C3A	100.93 (10)	H14A—C14—H14B	109.5
C12—C4—H4A	108.6	C13—C14—H14C	109.5
C5—C4—H4A	108.6	H14A—C14—H14C	109.5
C3A—C4—H4A	108.6	H14B—C14—H14C	109.5
C15—C5—C4	112.43 (11)	O15—C15—O16	124.01 (12)
C15—C5—C6	112.40 (11)	O15—C15—C5	125.88 (12)
C4—C5—C6	101.66 (10)	O16—C15—C5	110.11 (11)
C15—C5—H5A	110.0	O16—C16—C17	106.76 (12)
C4—C5—H5A	110.0	O16—C16—H16A	110.4
C6—C5—H5A	110.0	C17—C16—H16A	110.4
O10—C6—C6A	104.25 (10)	O16—C16—H16B	110.4
O10—C6—C5	101.29 (10)	C17—C16—H16B	110.4
C6A—C6—C5	108.24 (11)	H16A—C16—H16B	108.6
O10—C6—H6A	114.0	C16—C17—H17A	109.5
C6A—C6—H6A	114.0	C16—C17—H17B	109.5
C5—C6—H6A	114.0	H17A—C17—H17B	109.5
C6—C6A—C3B	100.08 (10)	C16—C17—H17C	109.5
C6—C6A—C7	116.41 (11)	H17A—C17—H17C	109.5
C3B—C6A—C7	100.52 (10)	H17B—C17—H17C	109.5
C6—C6A—H6AA	112.8		
			
C3—O2—C1—C9A	−59.58 (14)	C9A—O11—C7—C8	50.63 (11)
C6—O10—C3A—C3	−173.99 (10)	C9A—O11—C7—C6A	−58.08 (11)
C6—O10—C3A—C3B	−52.67 (11)	C6—C6A—C7—O11	−70.74 (13)
C6—O10—C3A—C4	60.53 (10)	C3B—C6A—C7—O11	36.17 (12)
C6A—C3B—C3A—O10	31.09 (12)	C6—C6A—C7—C8	−175.97 (11)
C9A—C3B—C3A—O10	−77.27 (12)	C3B—C6A—C7—C8	−69.05 (12)
C6A—C3B—C3A—C3	152.18 (11)	O11—C7—C8—C9	−32.10 (14)
C9A—C3B—C3A—C3	43.82 (14)	C6A—C7—C8—C9	73.94 (14)
C6A—C3B—C3A—C4	−74.76 (12)	C7—O11—C9A—C1	177.58 (10)
C9A—C3B—C3A—C4	176.89 (10)	C7—O11—C9A—C9	−51.00 (11)
C1—O2—C3—C3A	59.76 (14)	C7—O11—C9A—C3B	56.88 (11)
O10—C3A—C3—O2	64.99 (14)	O2—C1—C9A—O11	−63.19 (14)
C3B—C3A—C3—O2	−51.11 (15)	O2—C1—C9A—C9	177.15 (12)
C4—C3A—C3—O2	179.91 (11)	O2—C1—C9A—C3B	51.15 (15)
O10—C3A—C4—C12	−163.65 (10)	C3A—C3B—C9A—O11	75.48 (12)
C3—C3A—C4—C12	74.92 (15)	C6A—C3B—C9A—O11	−33.62 (12)
C3B—C3A—C4—C12	−55.30 (14)	C3A—C3B—C9A—C1	−44.38 (15)
O10—C3A—C4—C5	−39.44 (11)	C6A—C3B—C9A—C1	−153.48 (11)
C3—C3A—C4—C5	−160.87 (11)	C3A—C3B—C9A—C9	−179.32 (11)
C3B—C3A—C4—C5	68.91 (12)	C6A—C3B—C9A—C9	71.58 (12)
C12—C4—C5—C15	−110.49 (13)	C7—C8—C9—C9A	−0.35 (15)
C3A—C4—C5—C15	125.24 (11)	O11—C9A—C9—C8	32.70 (14)
C12—C4—C5—C6	129.10 (11)	C1—C9A—C9—C8	157.22 (13)
C3A—C4—C5—C6	4.83 (12)	C3B—C9A—C9—C8	−73.26 (14)
C3A—O10—C6—C6A	54.98 (11)	C13—O13—C12—O12	3.4 (2)
C3A—O10—C6—C5	−57.36 (10)	C13—O13—C12—C4	−177.31 (11)
C15—C5—C6—O10	−89.24 (12)	C5—C4—C12—O12	−9.39 (19)
C4—C5—C6—O10	31.18 (12)	C3A—C4—C12—O12	107.12 (15)
C15—C5—C6—C6A	161.48 (10)	C5—C4—C12—O13	171.36 (10)
C4—C5—C6—C6A	−78.09 (12)	C3A—C4—C12—O13	−72.14 (13)
O10—C6—C6A—C3B	−34.93 (12)	C12—O13—C13—C14	−143.18 (13)
C5—C6—C6A—C3B	72.31 (12)	C16—O16—C15—O15	−1.7 (2)
O10—C6—C6A—C7	72.24 (13)	C16—O16—C15—C5	178.70 (11)
C5—C6—C6A—C7	179.48 (10)	C4—C5—C15—O15	6.3 (2)
C3A—C3B—C6A—C6	2.12 (12)	C6—C5—C15—O15	120.28 (15)
C9A—C3B—C6A—C6	118.07 (10)	C4—C5—C15—O16	−174.16 (11)
C3A—C3B—C6A—C7	−117.38 (11)	C6—C5—C15—O16	−60.17 (14)
C9A—C3B—C6A—C7	−1.44 (12)	C15—O16—C16—C17	177.50 (13)

### Hydrogen-bond geometry (Å, º)

**Table:**

*D*—H···*A*	*D*—H	H···*A*	*D*···*A*	*D*—H···*A*
C3—H3*A*···O13	0.99	2.58	3.1604 (17)	118
C9—H9*A*···O13^i^	0.95	2.47	3.1692 (17)	131
C13—H13*A*···O2^ii^	0.99	2.58	3.1905 (17)	120
C13—H13*B*···O10^ii^	0.99	2.41	3.2193 (17)	139
C14—H14*C*···O15^i^	0.98	2.64	3.5669 (19)	158

Symmetry codes: (i) −*x*+1, *y*−1/2, −*z*+1/2; (ii) *x*, −*y*+1/2, *z*−1/2.
